# Participation in and attitude towards the national immunization program in the Netherlands: data from population-based questionnaires

**DOI:** 10.1186/1471-2458-12-57

**Published:** 2012-01-20

**Authors:** Liesbeth Mollema, Nancy Wijers, Susan JM Hahné, Fiona RM van der Klis, Hendriek C Boshuizen, Hester E de Melker

**Affiliations:** 1 Centre for Infectious Disease Control Netherlands, RIVM, Bilthoven, The Netherlands; 2 Department of Earth and Life Science, VU University Amsterdam, Amsterdam, The Netherlands; 3 ICT/Expertise Centre for Methodology and Information Service, RIVM, Bilthoven, The Netherlands; 4 National Institute for Public Health and the Environment, Centre for Infectious Disease Control Netherlands, Epidemiology and Surveillance Unit, P.O. Box 1, 3720 BA, Bilthoven, The Netherlands

## Abstract

**Background:**

Knowledge about the determinants of participation and attitude towards the National Immunisation Program (NIP) may be helpful in tailoring information campaigns for this program. Our aim was to determine which factors were associated with nonparticipation in the NIP and which ones were associated with parents' intention to accept remaining vaccinations. Further, we analyzed possible changes in opinion on vaccination over a 10 year period.

**Methods:**

We used questionnaire data from two independent, population-based, cross-sectional surveys performed in 1995-96 and 2006-07. For the 2006-07 survey, logistic regression modelling was used to evaluate what factors were associated with nonparticipation and with parents' intention to accept remaining vaccinations. We used multivariate multinomial logistic regression modelling to compare the results between the two surveys.

**Results:**

Ninety-five percent of parents reported that they or their child (had) participated in the NIP. Similarly, 95% reported they intended to accept remaining vaccinations. Ethnicity, religion, income, educational level and anthroposophic beliefs were important determinants of nonparticipation in the NIP. Parental concerns that played a role in whether or not they would accept remaining vaccinations included safety of vaccinations, maximum number of injections, whether vaccinations protect the health of one's child and whether vaccinating healthy children is necessary. Although about 90% reported their opinion towards vaccination had not changed, a larger proportion of participants reported to be less inclined to accept vaccination in 2006-07 than in 1995-96.

**Conclusion:**

Most participants had a positive attitude towards vaccination, although some had doubts. Groups with a lower income or educational level or of non-Western descent participated less in the NIP than those with a high income or educational level or indigenous Dutch and have been less well identified previously. Particular attention ought to be given to these groups as they contribute in large measure to the rate of nonparticipation in the NIP, i.e., to a greater extent than well-known vaccine refusers such as specific religious groups and anthroposophics. Our finding that the proportion of the population inclined to accept vaccinations is smaller than it was 10 years ago highlights the need to increase knowledge about attitudes and beliefs regarding the NIP.

## Background

The National Immunization Program (NIP) in the Netherlands currently offers children vaccinations against twelve infectious diseases (diphtheria, poliomyelitis, pertussis, tetanus, *Haemophilus influenzae *type b, meningococcal group C disease, measles, mumps, rubella, hepatitis B, pneumococcal disease, and cervical cancer caused by human papilloma virus). All children below the age of 13 years are eligible to receive vaccinations included in the NIP. Routine vaccination started in 1957, is nonmandatory and free of charge. Incidence rates of nearly all diseases targeted by the Dutch NIP have been reduced successfully. To maintain this success, continued high coverage and trust in vaccination by the population is of the utmost importance. Vaccination coverage for infants ranged from 94.5% for diphtheria, pertussis, tetanus and polio (DTaP-IPV) to 96.0% for measles, mumps and rubella (MMR) (cohort 2005; reporting year 2008). The coverage for toddlers and school-aged children was over 90% for each of the vaccinations (cohort 2002 and 1997 respectively; reporting year 2008) [1]. Although vaccine coverage in infants is high [1], previous studies have identified groups who (in part) refuse vaccination. One group consists of members of Reformed Congregations who believe that vaccination is contrary to the providence of God [2]. This group is at risk for epidemics as a result of sociogeographical clustering, which has been observed for polio, measles, mumps and rubella [3-6]. Another group consists of anthroposophics who believe that experiencing some childhood diseases may contribute to strengthening body and mind [2]. Anthroposophics are scattered throughout the Netherlands, but clustering in anthroposophic schools is present. The increased risk of epidemics has been observed for measles; in 2008, an outbreak occurred at several anthroposophic schools in the Netherlands [7] and also in anthroposophic communities abroad [8,9].

In addition, other less well-identified groups may exist that have doubts concerning the risks and benefits of vaccination. Paulussen et al. [10] observed that 81% of parents reported not having thought about the risks and benefits of vaccination thoroughly before making the decision. The authors hypothesized that these parents could easily be influenced by negative publicity about vaccination. In this study, we try to obtain more knowledge about the determinants of participation and attitude towards the NIP, which might be helpful in tailoring information campaigns for this program. In addition, over the course of a 10 year period, we were able to analyze possible changes in the opinion on vaccination during the preceding 5 years. In the Netherlands two nationwide seroprevalence surveys were performed, one in 1995-96, the other in 2006-07. Questionnaire data from these surveys have been used for the analyses.

## Methods

### Study Population

#### Sampling method

**Figure 1:**
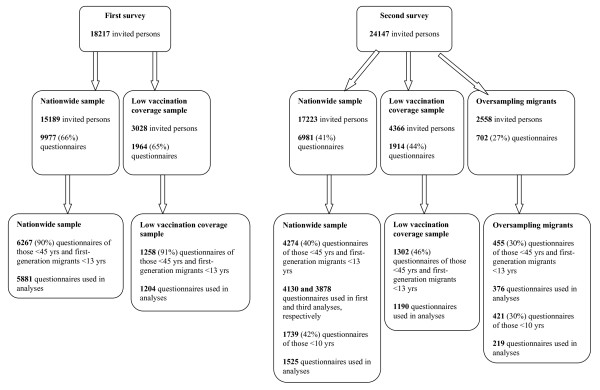
**Overview of the number of invited persons and the number of questionnaires (response rates) per sample and per survey**.

Two independent population-based cross-sectional serosurveillance surveys were carried out in the Netherlands between October 1995 and December 1996 (the first survey) and between February 2006 and June 2007 (the second survey) to establish a serum bank for the general population. The serum bank was to be used to estimate age-specific antibody levels against all vaccine preventable diseases in the (future) NIP and also of other diseases. Both surveys had a similar design, which has been described previously [11,12]. Briefly, the Netherlands were divided into five geographical regions of approximately equal population size. In each of the five regions a random sample of eight municipalities was drawn, proportional to the number of inhabitants. Within each municipality, an age-stratified sample (0, 1-4, 5-9, . . ., 75-79 years) of 380-500 persons (males and females) was drawn from the municipal health registers. In addition to the nationwide sample (NS), an age-stratified sample (similar as for the NS) from eight municipalities with low vaccination coverage (LVC) was drawn to assess the seroprevalence of groups of persons in sociogeographic clusters who refuse vaccination for religious reasons (known locally as the "Dutch Bible Belt"). The municipalities in the LVC sample were chosen on the basis of consistently low vaccination coverage (in the first survey for DTP-IPV for the years 1982-1993 and in the second survey for MMR and DTaP/IPV for birth cohorts 1997-2001) and the condition of representation of several provinces. The Dutch Bible Belt stretches from the southwest of the Netherlands to just above the centre in the northeast. In the second survey (2006-07), an extra sample from non-Western migrants was drawn from 12 of the 40 municipalities in the NS. We distinguished twelve migrant groups according to country of birth (1. Morocco and Turkey, 2. Suriname, Aruba and Netherlands Antilles and 3. Other non-Western countries), age (0-9 years, 10-49 years and 50-79 years) and first and second generation (only for 0-9 year-olds). A first-generation migrant was defined as somebody who was born abroad, immigrated to the Netherlands and whose parents (one or both) were born abroad. A second-generation migrant was defined as somebody who was born in the Netherlands and whose parents (one or both) were born abroad. The distribution of migrants per degree of urbanization (i.e., the number of addresses per km^2^) in the Netherlands was used to select the municipalities in which the oversampling of migrants took place. Figure 1 shows the number of invited persons per sample and per survey.

#### Approaching participants

Eligible persons received an invitation package by mail two weeks prior to the prescheduled appointment time for blood donation. This included the invitation letter, a brochure containing information on the survey, a questionnaire and an informed consent. Eligible persons were requested to donate a blood sample, to complete a questionnaire and to provide immunization records. All invited persons were approached by phone by a call centre to ask if they were willing to participate, to answer their questions and to remind them of the survey. When individuals refused to participate, they were asked to complete the questionnaire and if they also refused this, to answer some questions for the nonresponse survey (by telephone or mail). All participants gave signed informed consent.

### Questionnaire

The questionnaires were self-administered. In the first survey, three questionnaires were used for the following age groups: 0-11 years (42 questions); 12-16 years (42 questions); and 17-79 years (51 questions). The questionnaires were similar in content except that the questionnaire for 17 to 79-year-olds contained questions on sexual activities, alcohol and smoking. In the case of children below 12 years of age, parents mostly filled in the questionnaire.

In the second survey, two questionnaires were used for the following age groups: 0-14 years (50 questions) and 15-79 years (57 questions). The questionnaires were similar in content except that the questionnaire for 0 to 14 year-olds contained statements regarding vaccination and the questionnaire for those above 15 years of age contained questions on sexual activities. In the case of children below 15 years of age, parents mostly filled in the questionnaire.

Both in the first and second survey, the questionnaire covered topics such as age, gender, educational level, religion, number of persons in household, health status of the participant, participation in the NIP and whether their opinion on vaccination had changed with respect to the 5 years preceding the survey. Religion was categorised as followed: 1. High NIP refusers, low vaccination coverage, also called "Reformed Congregations" (i.e., Reformed Congregations, Reformed Congregations in the Netherlands, Old Reformed Congregations); 2. Moderate NIP refusers, intermediate vaccination coverage, also called "Reformed Bonders" (i.e., Restored Reformed Church and Reformed Bond); 3. Low NIP refusers, relative high vaccination coverage, also called "other specific Protestant Church" (i.e., other specific Protestant Christian religious communities); 4. Catholics; 5. Muslims; 6. Other religion (i.e., religions included see text below tables); 7. No religion. The questions on the above topics were asked in the same way in both surveys.

The questions on net monthly household income and "whether any of the following ideas influenced the participants'/parents' opinion on vaccination" (answer categories: anthroposophy (i.e., a spiritual philosophy founded by Rudolf Steiner), homeopathy (i.e., form of alternative medicine), other alternative medicine, religion, other, or none of those ideas) were only asked in the second survey. The last answer category "none of those ideas" was left out the analyses.

Only in the second survey, parents of children aged below 15 years were asked to answer the following question: "How many injections are acceptable for your child?" (answer categories: 0, 1, 2, 3, 4, any number is acceptable), and to agree or disagree with the following statements regarding vaccination: a) "Childhood vaccinations are useful for the protection of the health of my child"; b) "There is absolutely no need to vaccinate healthy children against childhood diseases"; c) "I doubt the safety of childhood vaccinations"; d) "The immune system of my child does not develop well due to vaccination"; e) "Childhood vaccinations are useful for the protection of the health of others". The answer categories for the vaccination statements were: "strongly agree"; "agree"; "neutral"; "disagree"; and "strongly disagree". Table 1 shows the questions regarding participation in the NIP, intention to accept any remaining vaccinations and changes of opinion regarding vaccination. Depending on the municipality, each participant was assigned a degree of urbanization and a region based on data from Statistics Netherlands available at http://www.cbs.nl.

**Table 1:** Overview of question-and-answer categories regarding vaccination included in the analyses

Question	Answer categories	First survey, NS	First survey,	Second survey, NS^a^	Second survey,
			LVC		LVC
		N (%)	N (%)	N (%)	N (%)
1. Did you/does your child participate in the NIP?^b^	Yes	5951 (96)	992 (79)	4420 (94)	940 (73)
	No	143 (2)	242 (19)	141 (3)	330 (25)
	Don't know^c^	109 (2)	18 (2)	127 (3)	26 (2)
		*64^d^*	*6^d^*	*41^d^*	*6^d^*

2. Do you intend to accept any remaining vaccinations for your child?^e^	1. Surely yes	NA	NA	1418 (68)	291 (46)
	2. Yes			484 (23)	148 (23)
	3. Probably yes			75 (4)	35 (5)
	4. Unsure			28 (1)	13 (2)
	5. Probably not			13 (1)	24 (4)
	6. Surely not			7 (0)	82 (13)
	7. Not applicable^f^			52 (3)	45 (7)
				*83^d^*	*5^d^*

3. Has your/parents' opinion on vaccination changed with respect to the five years preceding the survey?^b^	1. No	5514 (90)	1101 (89)	3866 (83)	1086 (84)
	2. Yes, more inclined	517 (8)	112 (9)	311 (7)	81 (6)
	3. Yes, less inclined	128 (2)	28 (2)	147 (3)	61 (5)
	4. Don't know	NA	NA	315 (7)	66 (5)
		*108^d^*	*17^d^*	*90*^d^	*8^d^*

^a^Including oversampling of non-Western migrants in the NS of the second survey

^b^Including participants aged less than 45 years and first-generation migrants aged less than 13 years

^c^Category "Don't know" was omitted from the analyses

^d^Number of missing answers at a specific question

^e^Including participants aged less than 10 years

^f^Category "not applicable" was omitted from the analyses

### Data analyses

SAS version 9.2 (SAS Institute Inc., Cary, NC, USA) was used for all analyses. Variable selection was based only on the complete cases, i.e., those without missing answers to questions. Below we consider the data analyses per subject.

### Nonparticipation in the NIP

Factors determining nonparticipation in the NIP (question 1, Table 1) were studied only in the NS of the second survey, including the oversampling of migrants. We included only participants aged less than 45 years of age and first-generation migrants aged less than 13 years at the time of participation in the study, i.e., those who could have participated in the NIP. People who reported not knowing whether they had participated in the NIP were excluded. To take into account the sample design, we adjusted all test determinants for age, gender, ethnicity, region and degree of urbanization. Determinants with a *P *value < = 0.05 from these analyses were included in the multivariate analyses. A determinant remained in the model if the *P *value was < = 0.05. The crude and adjusted odds ratios (ORs) and 95% confidence intervals (CIs) were calculated.

For several determinants associated with nonparticipation in the NIP the population attributable fraction (PAF) was estimated using the following equation: the probability at nonparticipation in the exposed population (i.e., including the group who participates less in the NIP compared to the reference group) minus the probability at nonparticipation in the nonexposed population (i.e., not including the group who participates less in the NIP compared to the reference group), divided by the probability at nonparticipation in the exposed population, using logistic regression modelling [13]. The PAF is expressed as the percentage of reduction in the prevalence of nonparticipation in the NIP in the population that would result from taking away the exposure to nonparticipation in the NIP [14]. The prevalence of the exposure to nonparticipation in the NIP used in the PAF was calculated based on the population in the NS, applying survey weights in order to make the sample representative for the Dutch population in terms of age, gender, ethnicity, degree of urbanization and region. Rao-Wu-Yue bootstrapping was used to obtain 95% confidence intervals, taking the study design into account [15].

### Parents' intention to accept any remaining vaccinations for their child

Factors determining reduced parental acceptance of any remaining vaccinations for their child (question 2, Table 1) were studied only in the NS of the second survey, including the oversampling of migrants. We included only participants aged less than 10 years of age (i.e., eligible for vaccinations in the NIP). For the dependent variable, we combined the answer categories "Surely yes", "Yes", and "Probably yes" for simplicity and, because of small numbers, we combined the answer categories "Unsure", "Probably not", and "Surely not". The answer category "Not applicable" was omitted from the analyses. The analyses were similar to those conducted on determinants of nonparticipation in the NIP.

### Changes in the opinion on vaccination between the 2006-07 and 1995-96 surveys

We investigated whether participants in the 2006-07 survey were more or less inclined to accept vaccination than the participants were in the 1995-96 survey. Separate analyses were performed for the NS (without the oversampling of migrants in the 2006-07 survey to be comparable with the survey in 1995-96 for which no oversampling was performed) and the LVC sample. For this purpose we analyzed the answers ("No", "Yes, more inclined", "Yes, less inclined") given to the question "Has your opinion on vaccination changed with respect to the 5 years preceding the survey?" (See also question 3 in Table 1). Participants less than 45 years old and first-generation migrants less than 13 years old at the time of participation in the study were included in the analyses. Note, migrants who participated in the NS of both surveys were included in the analyses. For the NS the following possible confounders were tested whether they influenced the variable sample (i.e., NS in the second survey versus NS in the first survey): educational level, religion and NIP participation, adjusted for age, gender, ethnicity, degree of urbanization and region. For the LVC sample the following possible confounders were tested whether they influenced the variable sample (i.e., LVC sample in the second survey versus LVC sample in the first survey): educational level, religion and NIP participation, adjusted for age and gender. Thereafter, a multivariate model was constructed by adding to the existing model the confounder that had the most influence on the coefficient of the variable sample. This process was repeated until no confounder remained that caused a change of more than 5% in the coefficient of the sample. The crude and adjusted ORs for the determinant sample and 95% CIs were calculated.

## Results

### Study population

Figure 1 shows the number of questionnaires obtained (response rates) per sample and per survey. Note, the numbers of questionnaires taken into account in the analyses were lower due to missing values or omitting certain answer categories. A relatively large number of the parents of migrant children less than 10 years of age did not fill in whether they agreed or disagreed with the statements regarding vaccination. Median age and interquartile range for the NS (including oversampling migrants in the second study) and the LVC sample was 31 (6-54) and 30 (6-54) in the first survey and 30 (8-55) and 25 (5-53) in the second survey, respectively. The proportion of males to females in the NS (including oversampling migrants in the second study) and the LVC sample was 0.9 and 0.9 in the first survey and 0.8 and 1.0 in the second survey, respectively. Table 1 shows the numbers and percentages of participants per answer category for the three questions used in the analyses in the first and second survey for the NS (including the oversampling of migrants in the second survey) and LVC sample separately.

### Nonparticipation in the NIP

The first column in Table 2 gives the numbers of participants per determinant and the second column shows the percentage of participants/parents who reported not to have participated in the NIP. Gender, degree of urbanization, region, household size, homeopathic, other alternative medicine and other beliefs were not significantly associated with reported participation in the NIP at univariate level (column 3, Table 2) and religious beliefs were no longer significant at multivariate level. The multivariate analyses showed that participants aged 10-29 years, who had a low income, who had a low educational level, who were of non-Western descent, who were members of Reformed Congregations or Reformed Bonders or who had anthroposophic beliefs regarding vaccination participated less in the NIP compared to the reference categories (column 4, Table 2).

**Table 2:** Potential determinants for reporting not to have participated in the NIP^a ^(N = 4506) and population attributable fractions (PAF)

Variable (N)	Reported	Crude^b^	Adjusted	PAF in %
	nonparticipation in the NIP in %	OR (95% CI)	OR (95% CI)	(95% CI)
**Age (yrs)**				

0-4 (1385)	3	1.7 (0.8-3.7)	1.4 (0.6-3.1)	

5-9 (660)	7	2.7 (1.2-6.0)	2.0 (0.9-4.5)	

10-29 (1470)	3	3.0 (1.4-6.2)	2.3 (1.1-4.9)	

30-44 (991)	1	Reference	Reference	

**Gender**				

Men (2051)	3	Reference	Reference	

Women (2455)	3	1.1 (0.8-1.6)	1.1 (0.7-1.5)	

**Ethnicity**				

Indigenous Dutch (3640)	2	Reference	Reference	

Other Western (226)	2	1.4 (0.5-3.5)	1.4 (0.5-3.8)	

Moroccan and Turkish (200)	19	11.7 (6.8-20.1)	7.1 (2.5-20.1)	21.5 (4.3-36.7)

Antilles and Aruba and Surinam (223)	6	3.1 (1.5-6.1)	3.3 (1.5-7.1)	4.4 (-1.0-11.7)

Other non-Western (217)	8	4.5 (2.4-8.2)	3.3 (1.6-7.1)	6.1 (-1.2-14.4)

**Religion**				

Reformed Congregations (59)	17	12.5 (5.2-30.1)	15.2 (6.1-37.8)	7.4 (0.1-18.4)

Reformed Bonders (129)	6	4.2 (1.7-10.4)	4.8 (1.9-12.1)	5.2 (0.1-13.1)

Other specific Protestant Church (805)	2	Reference	Reference	

Catholics (1183)	1	0.8 (0.4-1.6)	0.8 (0.4-1.6)	

Muslims (281)	16	1.4 (0.6-3.8)	1.2 (0.5-3.3)	

Other religion^c ^(204)	7	2.2 (1.0-4.9)	1,7 (0.7-3.9)	

No religion (1845)	2	0.8 (0.4-1.5)	0.7 (0.4-1.4)	

**Degree of urbanization**				

Very high (963)	6	Reference	Reference	

High (1998)	2	0.8 (0.5-1.3)	0.6 (0.4-1.0)	

Moderate (570)	2	0.9 (0.4-1.9)	0.6 (0.3-1.4)	

Low (452)	2	1.1 (0.4-3.3)	1.3 (0.4-4.2)	

Very low (523)	3	1.1 (0.6-2.2)	1.1 (0.5-2.2)	

**Region**				

Northeast (1023)	3	Reference	Reference	

Central (843)	2	0.8 (0.3-1.9)	0.6 (0.2-1.6)	

Northwest (1017)	5	1.0 (0.6-1.8)	0.8 (0.5-1.5)	

Southwest (808)	4	1.2 (0.6-2.3)	1.0 (0.5-1.9)	

Southeast (815)	2	0.6 (0.3-1.2)	0.7 (0.3-1.5)	

**No. of persons in household**				

1 (203)	2	0.8 (0.2-2.7)		

2 (516)	1	0.6 (0.3-1.3)		

3 (990)	3	1.2 (0.7-1.9)		

4 (1718)	2	Reference		

> 4 (1079)	5	1.4 (0.9-2.1)		

**Educational level^d^**				

Low (317)	13	2.1 (1.2-3.7)	2.5 (1.3-4.6)	13.7 (-1.2-29.7)

Moderate (2123)	3	1.1 (0.7-1.7	1.4 (0.9-2.2)	

High (2066)	2	Reference	Reference	

**Net monthly household income^e^**				

Low (569)	9	2.1 (1.1-4.1)	1.9 (1.0-4.0)	13.1 (-8.1-32.1)

Moderate (2051)	2	1.0 (0.5-1.8)	0.8 (0.4-1.6)	

High (936)	2	Reference	Reference	

Won't answer/missing answer (950)	2	0.97 (0.5-1.9)	0.9 (0.4-1.9)	

**Anthroposophy**				

Yes (46)	28	27.0 (12.8-57.0)	39.2 (17.5-87.9)	8.4 (2.3-17.6)

No (4460)	3	Reference	Reference	

**Homeopathy**				

Yes (75)	5	2.6 (0.9-7.5)		

No (4431)	3	Reference		

**Other alternative medicine**				

Yes (36)	8	2.6 (0.7-9.3)		

No (4470)	3	Reference		

**Religious belief**				

Yes (82)	17	9.5 (4.8-18.6)		

No (4424)	3	Reference		

**Other ideas influencing vaccination**				

Yes (189)	3	1.4 (0.6-3.3)		

No (4317)	3	Reference		

^a^The reference category is reporting "yes" at the question whether they (had) participated in the NIP

^b^Crude odds ratios were adjusted for age, gender, ethnicity, degree of urbanization and region

^c^Other religion included: Judaism, Buddhism and Hinduism

^d^Low educational level included no education and primary education; middle educational level included junior technical school, lower general secondary education and intermediate vocational education; and high educational level included senior/higher secondary education, pre-university education and university

^e^Net monthly income was categorized as low (less than € 1.150, which is less than € 1.167 by Statistic Netherlands); middle (€ 1.151-€ 3.050, which is € 1.168-€ 2.917 by Statistic Netherlands); high (more than € 3.051, which is more than € 2.918 by Statistic Netherlands); and a won't answer/missing answer category was included. Statistics Netherlands available at www.cbs.nl

By changing the ethnicity of Moroccan or Turkish participants into indigenous Dutch a higher population attributable fraction (21.5%) was observed than by changing low educational level into high educational level (13.7%) or low net monthly household income into high income (13.1%). In column 5 of Table 2 the PAFs of the various determinants are given.

### Parents' intention to accept any remaining vaccinations for their child

The proportion of parents who reported their child participated in the NIP was 96%. The first column in Table 3 gives the numbers of participants per determinant and the second column shows the percentages of parents who reported less likely to accept any remaining vaccinations for their child. In the univariate analysis gender, degree of urbanization, region, religion, net monthly household income, educational level, household size, own health and proposition "vaccination protects health others" were not significantly associated with parents' intention to accept any remaining vaccinations for their child (column 3, Table 3). Age, other beliefs, critical group (i.e., anthroposophy/homeopathy and other alternative medicine), religious beliefs and proposition "immune system does not develop well due to vaccination" were no longer significant at multivariate level.

**Table 3:** Potential determinants for parents less likely to accept any remaining vaccinations for their child^a ^(N = 1744)

Variables (N)	Parents who reported less likely to accept any remaining vaccinations for their child in %	Crude^b ^OR	Adjusted OR (95% CI)
		(95% CI)	
**Age (yrs)**			

0-4 (1237)	1	0.4 (0.2-0.9)	0.7 (0.3-1.9)

5-9 (507)	4	Reference	Reference

**Gender**			

Men (875)	2	Reference	Reference

Women (869)	2	1.2 (0.6-2.4)	0.6 (0.2-1.5)

**Ethnicity**			

Indigenous Dutch (1315)	1	Reference	Reference

Other Western (85)	1	0.7 (0.1-5.7)	1.5 (0.1-15.7)

Moroccan and Turkish (94)	7	3.7 (1.3-11.0)	1.7 (0.4-8.1)

Antilles and Aruba and Surinam (144)	2	1.0 (0.3-4.1)	1.0 (0.2-6.7)

Other non-Western (106)	5	2.4 (0.8-7.1)	4.2 (1.1-16.5)

**Religion**			

Reformed Congregations (22)	5	2.1 (0.2-19.8)	

Reformed Bonders (53)	2	1.4 (0.2-13.1)	

Other specific Protestant Church (325)	2	Reference	

Other religion^c ^(679)	3	1.4 (0.5-3.8)	

No religion (665)	1	0.4 (0.1-1.5)	

**Degree of urbanization**			

Very high (391)	3	Reference	Reference

High (738)	1	0.7 (0.3-1.9)	0.8(0.2-3.0)

Moderate (231)	1	0.7 (0.2-2.9)	1.3 (0.2-8.0)

Low (181)	1	1.6 (0.1-19.5)	1.5 (0.1-35.5)

Very low (203)	4	2.3 (0.7-7.4)	4.5 (0.99-20.1)

**Region**			

Northeast (379)	1	Reference	Reference

Central (337)	1	0.7 (0.1-6.6)	0.9 (0.1-12.0)

Northwest (434)	4	2.0 (0.7-5.8)	3.1 (0.6-14.9)

Southwest (302)	2	1.9 (0.5-7.6)	1.0 (0.2-6.7)

Southeast (292)	2	2.0 (0.5-7.6)	5.7 (0.97-33.4)

**Educational level^d^**			

Low (77)	8	1.4 (0.4-5.4)	

Moderate (832)	2	1.0 (0.5-2.0)	

High (835)	2	Reference	

**Net monthly household income^e^**			

Low (137)	7	3.0 (0.7-11.9)	

Moderate (863)	2	1.5 (0.5-4.6)	

High (405)	1	Reference	

Won't answer/missing answer (339)	2	1.3 (0.4-4.9)	

**No. of persons in household**			

1-2 (42)	2	1.0 (0.1-8.7)	

3 (451)	1	0.6 (0.2-1.7)	

4 (800)	2	Reference	

> 4 (451)	3	1.3 (0.6-2.7)	

**Health status child**			

Excellent/very well (1196)	2	Reference	

Well/moderate/poor (548)	3	1.6 (0.8-3.3)	

**Anthroposophic/** **homeopathic/** **other alternative medicine beliefs**			

Yes (83)	13	13.4 (5.9-30.3)	

No (1661)	1	Reference	

**Religious belief**			

Yes (38)	11	6.8 (2.1-22.4)	

No (1706)	2	Reference	

**Other ideas influencing vaccination**			

Yes (93)	8	5.7 (2.3-14.1)	

No (1651)	2	Reference	

**Maximum no. of injections**			

0 (13)	54	110.7 (9.5- > ∞)	27.7 (1.6-486.6)

1 (307)	6	3.6 (0.4-29.1)	1.5 (0.1-16.5)

2 (1376)	1	0.4 (0.1-3.5)	0.2 (0.0-2.7)

> 2 and each number is acceptable (48)	2	Reference	Reference

**Vaccinations protect health own child**			

Strongly agree/agree (1659)	1	Reference	Reference

Neutral/disagree/strongly disagree (85)	25	36.7 (16.5-81.4)	9.2 (3.0-27.8)

**Vaccinating healthy children is not necessary**			

Strongly agree/agree (57)	19	39.4 (14.4-107.5)	6.1 (1.6-23.7)

Neutral (115)	12	17.7 (7.3-42.9)	4.0 (1.2-13.0)

Disagree/strongly disagree (1572)	1	Reference	Reference

**Doubt safety vaccinations**			

Strongly agree/agree (181)	12	15.6 (6.9-35.1)	3.5 (1.1-11.0)

Neutral (423)	1	1.0 (0.3-3.4)	0.4 (0.1-1.8)

Disagree/strongly disagree (1140)	1	Reference	Reference

**Immune system does not develop well due to vaccination**			

Strongly agree/agree (144)	13	15.8 (6.9-36.1)	

Neutral (376)	2	1.7 (0.6-4.7)	

Disagree/strongly disagree (1224)	1	Reference	

**Vaccinations protect health of others**			

Strongly agree/agree (1275)	2	Reference	

Neutral (369)	2	1.2 (0.5-2.8)	

Disagree/strongly disagree (100)	5	2.5 (0.9-7.1)	

^a^Reference category is the combination of answer categories "surely yes", "yes", and "probably yes"

^b^Crude odds ratios were adjusted for age, gender, ethnicity, degree of urbanization and region

^c^Other religion included: Judaism, Buddhism, Hinduism, Catholic and Muslims

^d^Low educational level included no education and primary education; middle educational level included junior technical school, lower general secondary education and intermediate vocational education; and high educational level included senior/higher secondary education, pre-university education and university

^e^Net monthly income was categorized as low (less than € 1.150, which is less than € 1.167 by Statistic Netherlands); middle (€ 1.151-€ 3.050, which is € 1.168-€ 2.917 by Statistic Netherlands); high (more than € 3.051, which is more than € 2.918 by Statistic Netherlands); and a won't answer/missing answer category was included. Statistics Netherlands available at www.cbs.nl

The multivariate analyses showed that parents of other non-Western descent, who expressed the opinion that there should be no injection at all, who disagreed (including reporting neutral) with the proposition that "childhood vaccinations protect the health of one's own child", who agreed or reported neutral towards the proposition that "vaccinating healthy children is not necessary" or who agreed with the proposition "doubting the safety of childhood vaccinations" were less likely to accept any remaining vaccinations for their child compared to the reference categories (column 4, Table 3).

### Changes in the opinion on vaccination between the 2006-07 and 1995-96 surveys

The first and second column of Table 4 shows the number and percentages of participants/parents who reported to be more or less inclined to vaccinate. In both surveys about 90% of the participants or parents reported they had not changed their opinion towards vaccination in the 5 years preceding the survey. In the 2006-07 survey, fewer participants in the NS reported they were more inclined to accept vaccination and more participants reported they were less inclined to accept vaccination compared to no change in their opinion (see column 4 in Table 4). The odds ratios were adjusted for age, gender, ethnicity, degree of urbanization, region and educational level.

**Table 4:** Comparing opinion on vaccination in the 2006-07 survey^a ^with the 1995-96 survey

Nationwide sample^b^	N (%)	Crude OR (95%CI)	Adjusted OR (95%CI)
**More inclined to accept vaccination**			

First survey	498 (8)	Reference	Reference

Second survey	268 (7)	0.8 (0.7-0.9)^c^	0.8 (0.7-0.9)^d^

**Less inclined to accept vaccination**			

First survey	122 (2)	Reference	Reference

Second survey	131 (3)	1.4 (1.1-1.9)^c^	1.3 (1.0-1.7)^d ^

**Low vaccination coverage sample^e^**			

**More inclined to accept vaccination**			

First survey	108 (9)	Reference	Reference

Second survey	76 (6)	0.7 (0.5-1.0)^f^	0.6 (0.5-0.9)^g^

**Less inclined to accept vaccination**			

First survey	27 (2)	Reference	Reference

Second survey	60 (5)	2.2 (1.4-3.5)^f^	2.0 (1.2-3.2)^g^

^a^The reference category is: "My opinion had not changed with respect to the 5 years preceding the survey"

^b^The number of participants in the NS in the first and second survey was 5881 and 3878, respectively

^c^Adjusted for age, gender, ethnicity, degree of urbanization and region

^d^Adjusted for age, gender, ethnicity, degree of urbanization, region and educational level

^e^The number of participants in the LVC sample in the first and second survey was 1204 and 1190, respectively

^f^Adjusted for age and gender

^g^Adjusted for age, gender and religion (categories are as in Table 3)

In the 2006-07 survey, fewer participants in the LVC sample reported they were more inclined to accept vaccination and more participants reported they were less inclined to accept vaccination compared to no change in their opinion (see column 4 in Table 4). The odds ratios were adjusted for age, gender and religion.

## Discussion

As expected, on the basis of high vaccination coverage for infants for the vaccine-preventable diseases in the NIP [1], the attitude towards vaccination was generally positive. Not surprisingly, we observed that members of Reformed Congregations, Reformed Bonders and those with anthroposophic beliefs participated less in the NIP than those with no religion or no anthroposohic beliefs. We also observed other groups who participated less in the NIP compared to the reference categories, including those of non-Western descent, with a low income and with a low educational level.

Of interest, the analyses of population-attributable fractions showed that with regard to nonparticipation in the NIP, particular attention ought to be given to individuals with Moroccan or Turkish ethnicity and those with low educational and income level. They seem to contribute in large measure to the rate of nonparticipation in the NIP, i.e., to a greater extent than well-known vaccine refusers such as specific religious groups and anthroposophics.

Lower NIP participation among those with a low educational level or income compared to those with a high educational level or income is consistent with the recent observation that in areas with low socioeconomic status (SES) compared to high SES the uptake of human papilloma virus (HPV) vaccination in the catch-up campaign in 2009 was relatively low [16]. Furthermore, in a systematic review by Brown et al. [17], a lower vaccination uptake was linked to lower parental education and income compared to higher parental education and income. In contrast, several other studies showed a more negative attitude/intention or higher prevalence of nonadherents in relation to future vaccinations among persons with a higher educational level compared to those with a lower educational level [18-20]. Similar to our finding that NIP participation was lower among non-Western migrants compared to indigenous Dutch, the uptake of HPV vaccination was found to be lower among non-Western ethnicities than among indigenous Dutch, in particular, individuals of Moroccan and Turkish descent [16]. Other studies have observed a higher vaccination uptake among persons of Moroccan and/or Turkish descent compared to indigenous Dutch [21,22]. In the systematic review by Brown et al. [17] both a higher and a lower vaccine uptake was observed for non-white ethnicity compared to white ethnicity. We hypothesized that the lower observed NIP participation might be caused by a potential language barrier, as the invitation letter and information on vaccines included in the NIP they receive at home are in Dutch. They have to visit the NIP website to read the information in their own language. An evaluation might be undertaken to determine whether offering non-Western migrants more written and oral information in their own language would result in a higher NIP participation in this group compared to the daily practice as was described above.

No associations for religion, income and educational level were found with parents' intentions to accept any remaining vaccinations for their child in contrast to our findings for NIP participation. However, a similar trend was observed. It might be that numbers were too low to observe an association as only a subset of the population was used. Univariately, anthroposophic/homeopathic or alternative medicine beliefs were associated with parents' intention to accept any remaining vaccinations for their child. After adding statements regarding vaccination concerning safety, maximum number of injections, protection of the health of one's own child and whether vaccinating healthy children is necessary, the effect was not significant anymore. These parental concerns have been identified previously in the literature as associated with vaccination behaviour [10,17,18,21,23-27]. The vaccination statement "no good development of immunity" has been found in literature [10] to be associated with vaccination behaviour. Univariately, a high odds ratio was observed with those less likely to accept any remaining vaccinations, but by adding the other vaccination statements in the multivariate model the effect was no longer significant. Among those with low SES or of Moroccan or Turkish descent, it was not possible to study which opinions on vaccination were associated with parents' intention to accept any remaining vaccinations for their child. More (in-depth) research would be needed to verify why they are participating less in the NIP and to better understand their attitude and beliefs regarding the NIP.

Some limitations of this study must be discussed. This research was part of a larger seroepidemiological study and therefore, the number of questions related to attitude towards vaccination was limited. We also could not rule out any influence by reporting bias. A short pilot for the questionnaire was performed. The calculation of a PAF presumes that the effect of the determinant that is changed on vaccination rates is a causal effect. Such causality can not be inferred from our cross-sectional study. Therefore the PAF should be interpreted as the magnitude of the improvement of rates of reporting participation in the NIP that is maximally possible by removing the exposure to nonparticipation in the NIP if the effect of this exposure would be causal. Furthermore, overall responses of those who filled in a questionnaire of 66% and 40% in the first and second survey respectively, made nonresponse bias possible. The availability of background information made it possible to weight the PAFs for the variables known to be related with nonresponse, e.g., age, gender, ethnicity, region and degree of urbanization [28]. However, nonresponse bias related to other factors could be present.

It might be worrisome that both in the NS and in the LVC sample, participants in 2006-07 reported to be less inclined to accept vaccination than in 1995-96. To maintain trust in the vaccination program, we need to monitor the acceptance of the NIP in a timely fashion and try to find reasons behind a more reluctant attitude. It is likely that doubts regarding the decision to have children vaccinated precede changes in behaviour; use of our monitoring system for vaccination coverage will therefore probably be less sensitive and too slow. Future research will therefore focus on developing a system to monitor the acceptance of vaccinations among parents and (child) health care professionals and to review blogs and forums about vaccination on the internet. This system could be used to develop novel and targeted interventions to increase vaccination acceptance.

## Conclusions

Most participants had a positive attitude towards vaccination, although some had doubts. Groups with a lower income or educational level or of non-Western descent participated less in the NIP than those with a high income or educational level or indigenous Dutch and have been less well identified previously. Particular attention ought to be given to these groups as they contribute in large measure to the rate of nonparticipation in the NIP, i.e., to a greater extent than well-known vaccine refusers such as specific religious groups and anthroposophics. Our finding that the proportion of the population inclined to accept vaccinations is smaller than it was 10 years ago highlights the need to increase knowledge about attitudes and beliefs regarding the NIP.

## Competing interests

The authors declare that they have no competing interests.

## Authors' contributions

LM contributed greatly to the design and performance of the second population-based seroprevalence study, performed the statistical analysis of this study, and drafted the manuscript. NW assisted in performing statistical analysis of this study, and in drafting the manuscript. SH contributed to the design of the second population-based seroprevalence study, assisted in performing the statistical analysis of this study, and helped to draft the manuscript. FvdK made a large contribution to the design and performance of the second population-based seroprevalence study, and helped to draft the manuscript. HB assisted in performing the statistical analyses and helped to draft the manuscript. HdM made a large contribution to the design and performance of the first population-based seroprevalence study, contributed to the design of the second population-based seroprevalence study and revised the article critically for important intellectual content. All authors read and approved the final manuscript.

## Pre-publication history

The pre-publication history for this paper can be accessed here:

http://www.biomedcentral.com/1471-2458/12/57/prepub
